# Machine Learning-Based Integration Unveils RNA Methylation Regulator-Related Immune-Derived Gene Signatures in Ruptured Intracranial Aneurysm

**DOI:** 10.3390/biomedicines14061254

**Published:** 2026-05-30

**Authors:** Yiwen Wu, Jie Qiao, Yuchun Liu, Xian Yu, Haifeng Wang, Jianmin Zhang, Yi Huang

**Affiliations:** 1 Department of Neurosurgery, Second Affiliated Hospital, School of Medicine, Zhejiang University, Hangzhou 310009, China; wuyiwen@zju.edu.cn (Y.W.); yux668@zju.edu.cn (X.Y.); 2 Department of Endocrinology and Metabolism, The First Affiliated Hospital of USTC, University of Science and Technology of China, Hefei 230000, China; qiaojie@zju.edu.cn; 3 Department of Neurosurgery, Ningbo Hospital, School of Medicine, Zhejiang University, Ningbo 315000, China; 21618235@zju.edu.cn (Y.L.); fyywanghaifeng10630@nbu.edu.cn (H.W.); 4 Ningbo Key Laboratory of Nervous System and Brain Function, The First Affiliated Hospital of Ningbo University, Ningbo 315000, China

**Keywords:** aneurysmal subarachnoid hemorrhage, RNA methylation, immune inflammation, machine learning, oncostatin M, single-cell RNA sequencing

## Abstract

**Background/Objectives**: Immune-inflammatory activation is a central feature of aneurysmal subarachnoid hemorrhage (aSAH), yet the epitranscriptomic mechanisms underlying this response remain insufficiently understood. This study aimed to investigate RNA methylation-associated immune dysregulation in aSAH and to identify potential biomarkers and signaling pathways. **Methods**: Four Gene Expression Omnibus datasets were analyzed to characterize RNA methylation regulator-related immune alterations in aSAH. Single-sample gene set enrichment analysis (ssGSEA), weighted gene co-expression network analysis (WGCNA), and intersection with ImmPort immune genes were used to identify candidate genes. A total of 159 machine learning combinations were evaluated for model construction and external validation. Two-sample Mendelian randomization, single-cell RNA sequencing (scRNA-seq), and CellChat analyses were further performed. Peripheral blood samples from patients with aSAH (*n* = 12) and matched healthy controls (*n* = 12) were used for total m6A quantification and quantitative real-time PCR (qRT-PCR) validation, while Western blotting and immunofluorescence were used to validate the protein expression of LIFR, GP130, IGF2BP2, and RBM15B. **Results**: Eleven RNA methylation regulators were differentially expressed between aSAH and controls in GSE122897. The WGCNA module most strongly associated with RNA methylation regulator-related scores was enriched in immune response and myeloid activation pathways. Intersection analysis identified 25 candidate immune-inflammatory genes associated with RNA methylation regulator-related transcriptional patterns. Among 159 algorithms, an XGBoost-LASSO pipeline selected oncostatin M (OSM) as the key variable, and the resulting RNA methylation regulator-related immune-derived gene signature (RMRIGS) showed good discrimination between aSAH and controls across training and validation cohorts. Mendelian randomization supported a protective association of genetically predicted *OSM* expression with subarachnoid hemorrhage risk (IVW OR = 0.66, *p* = 0.014). Single-cell analysis showed that *Osm* was predominantly enriched in infiltrating *Ccr2*+ macrophages, whereas *Lifr* and *Il6st* were broadly expressed in activated microglial subpopulations, indicating the presence of an *Osm* − (*Lifr* + *Il6st*) communication axis after SAH. Clinically, total m6A levels were increased in peripheral blood samples from patients with aSAH, and *OSM*, together with several RNA methylation regulators, was upregulated and associated with m6A-related changes. In experimental models, the protein expression levels of LIFR, GP130, IGF2BP2, and RBM15B were all increased after SAH-related stimulation. **Conclusions**: RNA methylation programs may be involved in immune dysregulation in aSAH. The OSM-centered RMRIGS was associated with disease status and may provide insight into the interaction between peripheral immune activation and post-SAH neuroinflammation. The potential involvement of the OSM–LIFR/GP130 signaling axis and its association with RNA methylation regulator-related alterations warrant further investigation.

## 1. Introduction

Subarachnoid hemorrhage (SAH) is a severe subtype of stroke that accounts for approximately 5% of all strokes [1]. Around 85% of non-traumatic SAH cases are due to rupture of an intracranial aneurysm causing aneurysmal SAH (aSAH), while other causes may be cerebrovascular malformations, moyamoya disease, cerebral venous thrombosis, pituitary strokes, and primary cerebral hemorrhage [2]. aSAH is a complex cerebrovascular disease characterized by severe systemic complications, with a global mortality rate of approximately 40% [3]. aSAH results in not only rapid, but also varied brain damage, including early brain injury (EBI), secondary brain injury, delayed cerebral ischemia (DCI), and long-term neurological dysfunction. Specifically, EBI is closely associated with the risk of delayed cerebral ischemia (DCI) and long-term morbidity [4]. The brain damage caused by aSAH is severe and irreversible, which contributes to the high rate of disability and mortality in patients with aSAH. Therefore, the prevention and treatment of aSAH remains a major challenge for neurosurgeons. Meanwhile, advances in microsurgical and endovascular techniques, including flow-diversion strategies such as the pipeline embolization device, have expanded treatment options for complex intracranial aneurysms; however, aneurysm rupture and post-aSAH complications remain major clinical challenges [5].

Saccular intracranial aneurysms usually develop over a long period, particularly at arterial bifurcations, under the combined influence of hemodynamic stress, hypertension, genetic susceptibility, vascular wall degeneration, extracellular matrix remodeling, and chronic inflammation. During aneurysm progression, loss of cellular components, degradation of the extracellular matrix, and progressive thinning of the aneurysm wall may gradually increase rupture susceptibility. Inflammatory and immune processes within the aneurysm wall can promote or accompany different phases of aneurysm development and rupture. After rupture, the pathological process further involves acute hemorrhage-induced systemic inflammation, neurovascular injury, and post-SAH neuroinflammation. The immune-inflammatory response to intracranial aneurysm rupture may involve many different inflammatory cells/factors, such as tumor necrosis factor (TNF), which can regulate the phenotype of smooth muscle cells and cause an increase in the expression of matrix metalloproteinase (MMP), which in turn damages the extracellular matrix and the vascular wall. The combination of inflammatory cells or factors leads to a sustained remodeling of the aneurysm wall that is unable to resist hemodynamic stresses and rupture [6]. The immune-inflammatory response is also an important mechanism of brain injury after aSAH and plays a pivotal role in the development of EBI and DCI [7]. Previous studies have demonstrated that neuroinflammation may interact with microvascular dysfunction to initiate brain injury and DCI after aSAH [8]. The immune-inflammatory response plays an important regulatory role in all aspects of aSAH development, so further studies on the correlation between inflammation and aSAH are expected to reveal new targets and ideas for the prevention of aneurysm rupture as well as the treatment of patients with aSAH.

RNA modification is an important post-transcriptional processing program that plays an influential regulatory role in a variety of physiological processes and disease progression. Currently, more than 170 RNA modifications have been identified in eukaryotes [9]. Among them, N6-methyladenosine (m6A) is one of the most abundant internal modifications in eukaryotic mRNA [10]. N6-methyladenosine modification of RNAs is involved in almost all processes involved in mRNA metabolism, including RNA transcription, translation, and degradation [11]. m6A modification is a dynamic and reversible process, which is catalyzed by m6A methyltransferases, removed by m6A demethylases, and recognized by m6A binding proteins [12]. It has been reported that m6A modification regulates the expression of immunoinflammatory-related genes and thus mediates inflammation in a variety of diseases, including metabolic diseases, autoimmune diseases, tumors, etc. [13]. m6A modification has also been reported to play a crucial role in key biological processes and pathogenesis of various neurological disorders, including Alzheimer’s disease [14], Parkinson’s disease [15], and gliomas [16]. In addition, m6A methylation modification levels have been reported to be dysregulated in intracranial aneurysms and associated with intracranial aneurysm development and rupture [17]. While epigenetic modifications are closely associated with immune-inflammatory responses, whether m6A specifically participates in the injury and inflammatory response of brain tissue after SAH has not yet been fully investigated. In addition to m6A modification, there are other types of RNA methylation, including N1-methyladenosine (m1A), 5-methylcytosine (m5C), and N7-methylguanosine (m7G). All of these RNA methylations have been reported to be involved in the regulation of inflammatory responses in different diseases, such as abdominal aortic aneurysm [18], idiopathic pulmonary fibrosis [19], and sepsis [20]. Therefore, investigating the relationship between RNA methylation regulator-related transcriptional programs and immune-inflammatory responses in aSAH may provide useful molecular clues.

Bioinformatics allows for the processing of genetic information and the mining of multi-omics data to discover key regulatory genes. This discipline is often used in clinical practice to analyze transcriptome-level changes and search for therapeutic targets [21]. In this study, we aimed to explore RNA methylation regulator-related immune-inflammatory alterations after aneurysmal rupture and aSAH using an integrated approach combining bioinformatics, machine learning, and experimental validation. We utilized 159 algorithm combinations to screen and build an RNA methylation regulator-related immune-derived gene signature (RMRIGS) based on the training dataset GSE122897. Because predictive associations alone cannot establish causality, we complemented our findings with two-sample Mendelian randomization (MR) to test whether genetically predicted OSM is linked to a reduced risk of aSAH. Importantly, immune infiltration analyses from bulk data converged on a prominent myeloid signal, with macrophage-related infiltration scores showing the largest shifts, which motivated the use of scRNA-seq to resolve cellular heterogeneity, pinpoint the dominant cellular source(s) of OSM, and infer OSM-associated intercellular communication networks. We then validated these computational findings through clinical m6A quantification and qRT-PCR in peripheral blood, together with in vivo and in vitro models to assess receptor axis-related protein changes and RNA methylation regulator-related alterations, ultimately aiming to provide candidate molecular clues for RNA methylation-associated immune-inflammatory alterations after aneurysmal rupture and aSAH.

## 2. Materials and Methods

### 2.1. Data Collection

**Figure 1:**
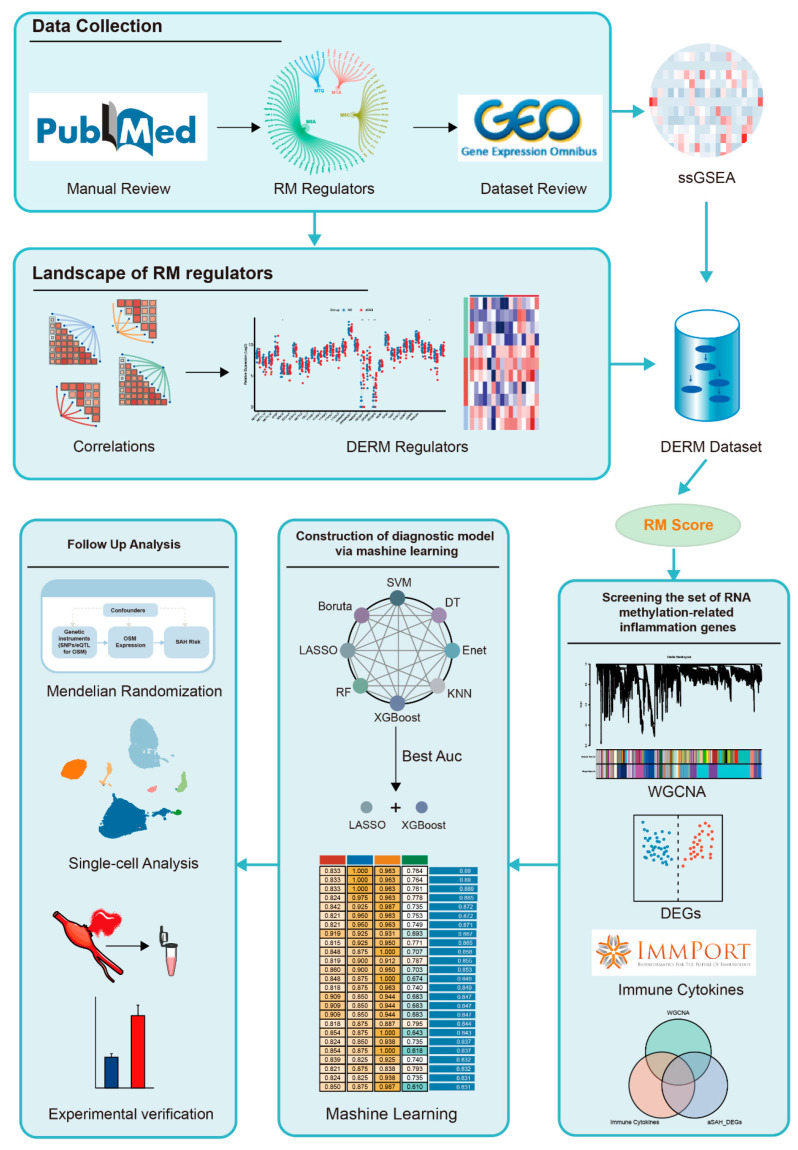
Workflow of our current work in aSAH. AUC, area under the curve; DEGs, differentially expressed genes; DERM, differentially expressed RNA methylation regulators; DT, decision tree; Enet, elastic net; KNN, k-nearest neighbor; LASSO, least absolute shrinkage and selection operator; WGCNA, weighted gene co-expression network analysis; RF, random forest; ssGSEA, single-sample gene set enrichment analysis; XGBoost, eXtreme Gradient Boosting.

The overall workflow of this study is depicted in Figure 1. Transcriptomic datasets related to intracranial aneurysm and aSAH were accessed from Gene Expression Omnibus (GEO, http://www.ncbi.nlm.nih.gov/geo, accessed at: 25 November 2025) database, including GSE122897, GSE54083, GSE15629 and GSE36791. Among them, GSE122897 was an RNA-seq dataset based on the GPL16791 (Illumina HiSeq 2500, Illumina, San Diego, CA, USA) platform. GSE54083 and GSE36791 were microarray datasets based on the GPL4133 (Agilent-014850 Whole Human Genome Microarray 4 × 44K G4112F [Feature Number version], Agilent Technologies, Santa Clara, CA, USA) platform and the GPL10558 (Illumina HumanHT-12 V4.0 expression, Illumina, San Diego, CA, USA) platform. GSE15629 was a microarray dataset based on GPL6224 (Affymetrix Human Gene 1.0 ST Array [transcript (gene) version], Affymetrix Inc., Santa Clara, CA, USA) platform. The RMRIGS model was constructed using the GSE122897 dataset and externally validated using the GSE54083, GSE15629, and GSE36791 datasets. The datasets were preprocessed by quantile normalization and log2 transformation to make them more comparable between samples. For Mendelian randomization (MR) analysis, the eQTL data for OSM (Ensembl ID: ENSG00000099985) were obtained from the OpenGWAS Project (OpenGWAS ID: eqtl-a-ENSG00000099985), based on the HG19/GRCh37 reference genome and a European population. The GWAS summary statistics for subarachnoid hemorrhage were obtained from the FinnGen R12 database (GWAS ID: finngen_R12_I9_SAHANEUR). For scRNA-seq analysis, publicly available mouse brain datasets were collected from GEO and the National Genomics Data Center. The sham dataset was obtained from GEO (GSM5319987), and the SAH datasets were obtained from OMIX (OMIX006611), including sham, SAH day 1, and SAH day 7 samples. These datasets were used to characterize the myeloid cell landscape and to explore OSM-related intercellular communication after SAH. These datasets represented different biological contexts and were used for distinct analytical purposes. Public human bulk transcriptomic datasets were used for feature discovery, model construction, and external validation; mouse SAH single-cell RNA-seq datasets were used to explore cell type heterogeneity and potential OSM-associated intercellular communication after experimental SAH; and human peripheral blood samples were used for exploratory validation of systemic immune-inflammatory and RNA methylation regulator-related changes. Detailed sample characteristics, biological contexts, analytical roles, and major limitations of each dataset and validation sample are summarized in Table S1.

### 2.2. Data Preprocessing and Differential Expression Analysis

The RNA read count data of GSE122897 were normalized by the Voom method and further analyzed using the Limma R package (version 3.48.3). The screening criteria for differentially expressed genes (DEGs) were log2FoldChange absolute value more than 1 and false discovery rate (FDR) less than 0.05. In addition, the GSE54083, GSE15629, and GSE36791 datasets were preprocessed using quantile normalization and log2 transformation when required.

### 2.3. Identification and Correlation Analysis of RNA Methylation Regulators

Overall, we selected four types of RNA methylation regulators according to recent publications [10,22,23], including m6A regulators, m5C regulators, m1A regulators, and m7G regulators. The RNA methylation regulator gene sets are listed in Supplementary Table S2. The expression profiles of the RNA methylation regulators were extracted from the GSE122897 dataset. Correlation analysis between the RNA methylation regulators was performed using Spearman’s correlation methods. The significantly differentially expressed RNA methylation regulators between aSAH and control samples were identified via Student’s *t*-test or Wilcoxon rank-sum test. The expression patterns of the differentially expressed RNA methylation regulators were visualized with a heatmap.

### 2.4. Calculation of RNA Methylation Regulator-Related Scores and Immune Characteristics

To characterize RNA methylation regulator-related transcriptional patterns in each sample, ssGSEA scores were calculated based on predefined RNA methylation regulator gene sets [24]. These scores were interpreted as regulator-expression-based transcriptional indices rather than direct measurements of RNA methylation abundance. In addition, we also evaluated the abundance of infiltrating immune cells for each sample using the ssGSEA algorithm. This approach was achieved by using the GSVA R package (version 1.40.1). Please refer to the Supplementary Table S3 for details of the gene sets marking 24 immune cells.

### 2.5. Weighted Correlation Network Analysis (WGCNA) and Screening for Relevant Immune Genes

Co-expression mRNA networks of GSE122897 were generated using the WGCNA algorithm. The criterion of scale-free networks was satisfied by calculating a suitable soft threshold β. We converted the weighted adjacency matrix into a topological overlap matrix (TOM) and converted the corresponding dissimilarity (1-TOM). To identify co-expression modules associated with RNA methylation-related transcriptional patterns, module eigengenes were correlated with m6A-, m5C-, m1A-, and m7G-related ssGSEA scores. Both positively and negatively correlated modules were considered, and the final candidate module was selected based on the consistency, statistical significance, and absolute strength of its associations with multiple RNA methylation regulator-related scores. Immune genes (Supplementary Table S4) were obtained from the ImmPort database (https://www.immport.org/home, accessed at: 18 October 2022) and then intersected with the differentially expressed genes and genes from the RNA methylation regulator-related candidate module of the GSE122897 dataset to screen candidate immune-inflammatory genes associated with RNA methylation regulator-related transcriptional patterns. The differential genes in the normal and aSAH groups were calculated using the limma package in R.

### 2.6. Enrichment Analysis

To determine the status of the most highly correlated module in WGCNA, we performed enrichment analyses by Gene Ontology (GO) and Kyoto Encyclopedia of Genes and Genomes (KEGG) to obtain its biological function. The above procedure was performed using the clusterProfiler, enrichGO, and enrichKEGG packages in R, and the results of the enrichment analysis were visualized using the ggplot2 package (version 3.3.5).

### 2.7. Integrated Machine Learning-Based Approaches to Identify RMRIGS

To identify the optimal diagnostic model, we integrated seven feature selection algorithms, including least absolute shrinkage and selection operator (LASSO), elastic net regression (Enet), decision tree (DT), random forest (RF), Boruta, support vector machine recursive feature elimination (SVM-RFE), and eXtreme Gradient Boosting (XGBoost), with seven machine learning classifiers, including LASSO, ridge regression (Ridge), support vector machine (SVM), k-nearest neighbor (KNN), RF, DT, and XGBoost, resulting in 159 algorithm combinations. The GSE122897 dataset was used as the training cohort. Considering the limited sample size and class distribution of the training cohort, model hyperparameters were optimized using 3-fold cross-validation within the training cohort. The final model was further evaluated in independent external validation cohorts to assess its generalizability. GSE54083, GSE15629, and GSE36791 were used as external validation cohorts. The area under the receiver operating characteristic curve (AUC) was used to evaluate model performance, and the average AUC across the training and external validation cohorts was used as the main criterion for model comparison. These procedures were mainly implemented using the mlr3 R package (version 0.11.0).

The final RMRIGS model was constructed using the XGBoost-LASSO pipeline. XGBoost was first used for feature prioritization, and LASSO regression was then applied for final variable selection. The RMRIGS score was calculated using the following formula:

RMRIGS score = 0.0795 × *OSM* expression

### 2.8. Mendelian Randomization (MR) Analysis

A two-sample Mendelian randomization (MR) analysis was performed to assess the potential causal effect of *OSM* expression on SAH risk. The exposure data were obtained from the *OSM* eQTL dataset in the OpenGWAS database (eqtl-a-ENSG00000099985; Ensembl ID: ENSG00000099985), and the outcome data were derived from the FinnGen R12 database (finngen_R12_I9_SAH). SNPs significantly associated with *OSM* expression (*p* < 5 × 10^−8^) were selected as instrumental variables and pruned for linkage disequilibrium (r^2^ < 0.001, 10,000 kb window). After harmonization, causal estimates were primarily obtained using the inverse-variance weighted (IVW) method and further evaluated using constrained maximum likelihood and model averaging (cML-MA), contamination mixture (ConMix), Mendelian randomization robust adjusted profile score (MR-RAPS), and debiased inverse-variance weighted (dIVW). Instrument strength was evaluated using the F-statistic, with F > 10 considered indicative of sufficient instrument strength. Heterogeneity was assessed using Cochran’s Q test, and horizontal pleiotropy was assessed using the MR-Egger intercept test and MR-PRESSO global test. A leave-one-out analysis was performed to evaluate whether the causal estimate was disproportionately influenced by any individual SNP.

### 2.9. Single-Cell RNA-Seq Analysis

Single-cell RNA-seq datasets from sham and SAH mouse brains were collected from public databases and processed using a standard Seurat workflow (version 5.3.1). Low-quality cells were filtered according to the number of detected genes, unique molecular identifiers, and mitochondrial gene percentage. After data normalization and identification of highly variable genes, batch effects between samples were corrected using the Harmony algorithm. Dimensionality reduction was performed by principal component analysis (PCA), followed by Uniform Manifold Approximation and Projection (UMAP) for visualization. Cell clusters were identified using a graph-based clustering method and annotated according to canonical marker genes.

To further characterize the heterogeneity of myeloid cells, the microglia/macrophage population was extracted and reclustered. Subclusters were annotated based on known marker genes, such as homeostatic microglia, activated microglia, inflammatory microglia, IFN microglia, proliferating microglia, and *Ccr2* macrophages. The expression patterns of *Osm* and RNA methylation-related regulators were then compared across cell types, subclusters, and experimental groups.

Cell–cell communication analysis was performed using the CellChat package (version 2.1.2) in R. Communication probabilities and ligand–receptor interactions were inferred separately for different groups, with a focus on OSM-related signaling. The *Osm* − (*Lifr* + *Il6st*) axis was further examined to identify potential communication between Ccr2 macrophages and microglial subpopulations.

Because the bulk transcriptomic discovery and clinical validation were based on human samples, whereas the single-cell RNA-seq datasets were derived from mouse SAH models, mouse gene symbols were mapped to their human orthologs when cross-species interpretation was required. Only genes with clear homologous annotations and direct relevance to the OSM-associated communication axis were interpreted across species. Specifically, mouse *Osm*, *Lifr*, *Il6st*, and *Ccr2* correspond to human *OSM*, *LIFR*, *IL6ST* and CCR2, respectively, *IL6ST* encodes GP130. Therefore, the mouse single-cell analysis was used mainly to infer the cellular source and potential recipient populations of OSM-related signaling after SAH, rather than to directly train or validate the human diagnostic model. The main mouse–human homologous genes interpreted in this analysis are summarized in Table S15.

### 2.10. RNA m6A Quantification and Quantitative Real-Time PCR (qRT-PCR)

Patients (≥18 years old, *n* = 12) diagnosed with aSAH between October 2021 and October 2022 at the Ningbo Hospital of Zhejiang University were recruited for this study. Patients with other diseases or comorbidities were excluded. In total, 12 sex- and age-matched healthy controls were also selected. Based on previous studies [25,26], we extracted total RNA from blood samples using TRIzol (Servicebio, Wuhan, China) and assessed RNA quality using NanoDrop 2000 spectrophotometer (Thermo Fisher Scientific, Waltham, MA, USA). The EpiQuik m6A RNA Methylation Quantification Kit (Epigentek, Farmingdale, NY, USA) was used to examine the m6A modification level of each patient. Briefly, 200 ng RNA was coated on test wells, followed by capture and detection antibody solutions. Subsequently, we determined the m6A level by detecting the absorbance at 450 nm (OD450) for each sample and calculated the m6A content from the standard curve. The steps of RNA extraction and quality detection of qRT-PCR were consistent with those of m6A methylation detection. We used Servicebio^®^RT First Strand cDNA Synthesis Kit to synthesize cDNA and detected relative RNA levels using the SYBR Green method. The relative expression of each gene was calculated as the power value (2^−△△C_t_^). The primer sequences used for RT–qPCR are available in Supplementary Table S5.

### 2.11. In Vivo and In Vitro Validation

C57BL/6 mice were used to establish the SAH model by endovascular perforation, and sham-operated mice were used as controls. Brain tissues were harvested at 3 and 7 days after SAH induction for Western blotting and immunofluorescence analyses. BV2 microglial cells were treated with 30 μM oxyhemoglobin (OxyHB) for 24 h to simulate the SAH environment in vitro. The protein expression levels of LIFR protein encoded by *LIFR*, GP130 protein encoded by *IL6ST*, IGF2BP2, and RBM15B were detected by Western blotting in both BV2 cells and mouse brain tissues, with β-actin used as the internal control. Immunofluorescence staining was further performed to assess the co-localization of IBA1 and GP130 in brain tissues.

### 2.12. Statistical Analysis

Statistical analysis was performed using R software (version 4.1.0). The ggpubr package (version 0.4.0) was used to generate group comparison plots. Comparisons between two groups were performed using Student’s *t*-test or the Wilcoxon rank-sum test, as appropriate. Comparisons among three groups were performed using the Kruskal–Wallis test. Correlations between continuous variables were assessed using Spearman’s correlation analysis. The m6A-regulator expression score was calculated as the mean of z-score-transformed qRT-PCR expression values of available m6A regulators and correlated with directly measured total m6A levels using Spearman’s correlation analysis. For clinical validation markers, two-sided Welch’s *t*-test, Hedges’ g effect size, and post hoc power analysis were additionally calculated. A *p*-value < 0.05 was considered statistically significant.

## 3. Results

### 3.1. Landscape of RNA Methylation Regulators Between Control and aSAH Groups

**Figure 2:**
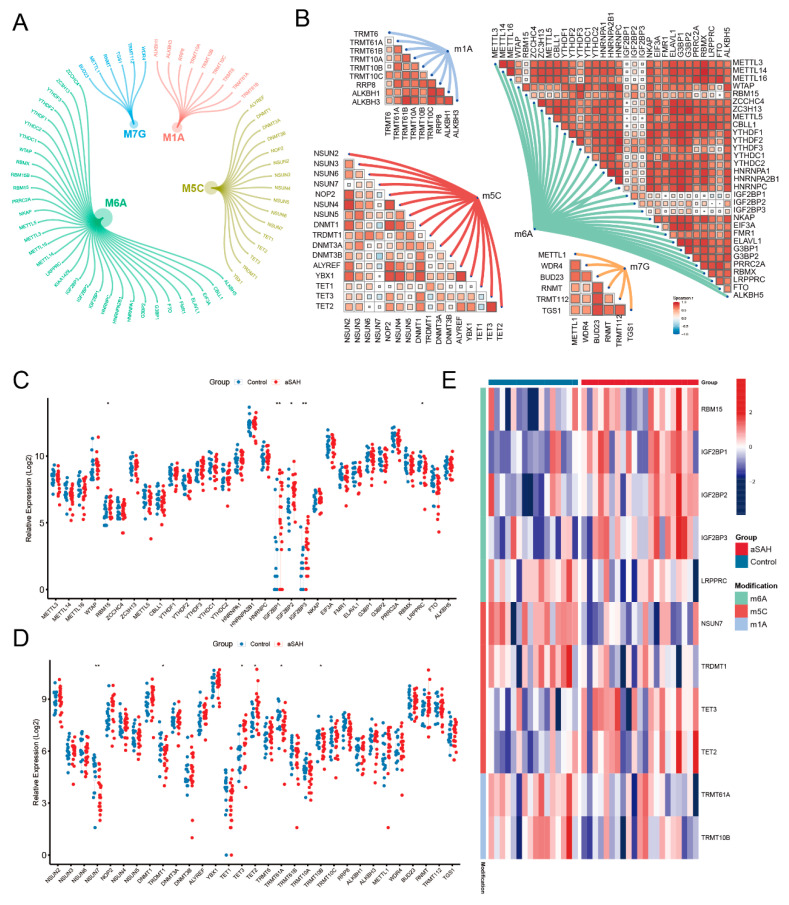
Landscape of RNA methylation regulators in aSAH. (**A**). Dendrogram summarizing 64 RNA methylation regulators. (**B**). Heatmap for the intrinsic correlation of different RNA methylation regulators, with positive correlation in red and negative correlation in blue. (**C**). Differential expression of 33 m6A regulatory factors between aSAH and controls. (**D**). Differential expression of 16 m5C factors, 9 m1A factors, and 6 m7G factors between aSAH and controls. (**E**). Heatmap for 11 differentially regulated factors between the two groups. * *p*-value < 0.05, ** *p*-value < 0.01.

We summarized four groups of recognized RNA methylation regulators (33 m6A factors, 16 m5C factors, 9 m1A factors, and 6 m7G factors) from previous publications (Figure 2A). To explore the association between different RNA methylation regulatory intrinsic factors, we conducted a transcriptome correlation study and found that there was a close correlation among the RNA methylation regulatory intrinsic factors (Figure 2B). Expression profiling showed that the expression levels of five m6A, four m5C, and two m1A regulators were significantly different between the aSAH group and the control group (Figure 2C,D). Among them, the expression of 4 m6A genes (*RBM15*, *IGF2BP1*, *IGF2BP2*, and *IGF2BP3*) and 2 m5C genes (*TET3*, *TET2*) were up-regulated. One m6A gene (*LRPPRC*), two m5C genes (*NSUN7*, *TRDMT1*) and two m1A genes (*TRMT61A*, *TRMT10B*) were down-regulated. Finally, the expression patterns of these 11 RNA methylation regulators were depicted in a heatmap (Figure 2E).

### 3.2. Identification of Genes Related to RNA Methylation Regulation by WGCNA and ssGSEA Association

**Figure 3:**
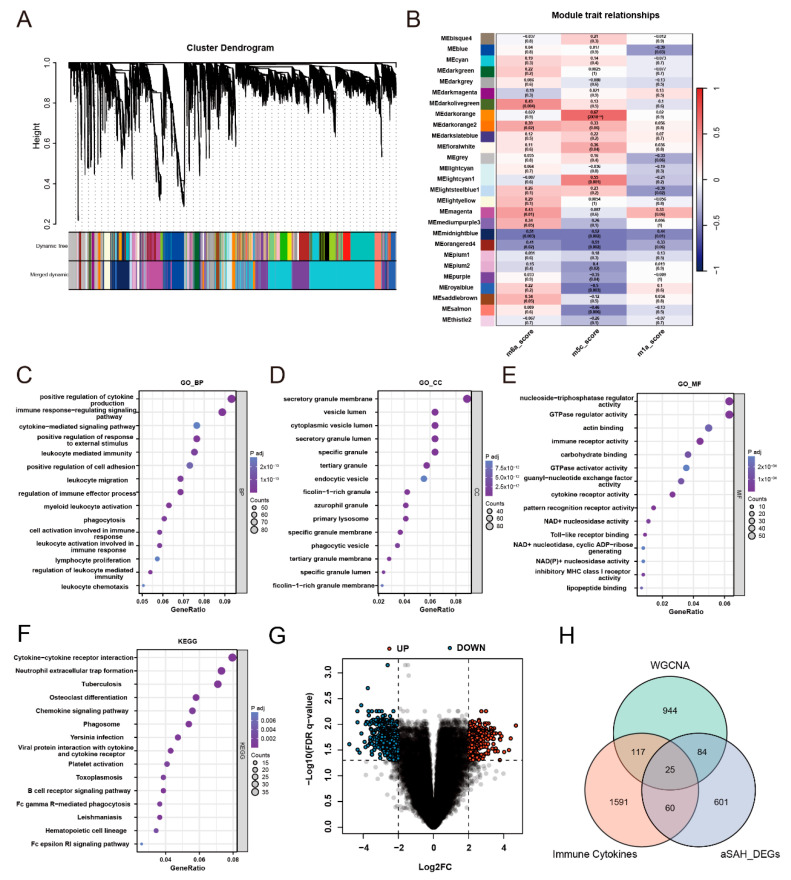
Primary filtering of RNA methylation-associated immunoinflammatory genes. (**A**). Gene clustering with co-expression module identification. (**B**). Heatmap of the correlation of co-expressed gene modules with m6A, m5C, and m1A, with numbers in the upper part of the module representing the correlation, and numbers in the lower part of the module in parentheses representing the *p*-value. (**C**–**F**). Top 15 enrichment results of midnight blue module genes in GO and KEGG. (**G**). Volcano plot of aSAH versus control DEGs. (**H**). Screening of 25 potential molecules for RNA methylation-associated immunoinflammation in aSAH by overlapping the DEGs of aSAH, WGCNA module-associated genes, and immunoinflammatory genes in the ImmPort database.

To obtain genes associated with RNA methylation regulation, the GSE122897 dataset was analyzed using weighted gene co-expression network analysis (WGCNA), which identified 26 non-gray gene modules (Figure 3A). The enrichment scores of m6A, m5C, and m1A were calculated for each sample using ssGSEA. Among all modules, the midnight-blue module showed significant and consistent correlations with multiple RNA methylation-related ssGSEA scores, including m6A (r = −0.51, *p* = 0.003), m5C (r = −0.52, *p* = 0.002), and m1A (r = −0.44, *p* = 0.01) (Figure 3B). Modules with positive correlations were also evaluated; however, the midnight-blue module was selected because it showed the most consistent association across three RNA methylation-related scores, rather than because of the negative correlation direction alone. These findings indicated that the midnight-blue module was closely associated with RNA methylation regulator-related transcriptional patterns and may represent an immune-inflammatory co-expression module in aSAH.

### 3.3. Enrichment of RNA Methylation-Related Module Gene Functions and Pathways

We further analyzed the biological significance of the midnight-blue gene module in the previous step, and GO analysis showed that this module may be involved in several biological processes, cellular components and molecular functions. The significant enrichment in immune response-regulating signaling pathways, myeloid leukocyte activation, secretory granule membrane, and immune receptor activation suggests that this module may be hypothesized as an immune-related gene module (Figure 3C–E).

In addition, in order to explore the possible functions of the genes involved in this module at the pathway level, the genes were enriched by the KEGG database. Neutrophil extracellular trap network formation was the most significantly enriched pathway, and cytokine–cytokine receptor interactions were enriched for the highest number of genes (Figure 3F). It is reasonable to hypothesize that RNA methylation is likely to play a biological role in aSAH genesis by influencing a variety of cytokines, particularly those associated with myeloid cells in immune cells.

### 3.4. Preliminary Screening of the RNA Methylation-Associated Immunoinflammatory Gene Set

Firstly, the GSE122897 dataset was used to screen aSAH differentially expressed genes (aSAH_DEGs). 264 genes were up-regulated and 507 genes were down-regulated compared with the control group (Figure 3G, Supplementary Table S6). In order to determine the expression of immune-inflammation-related molecules in aSAH, 1793 genes were obtained by searching the ImmPort database, which was associated with immune-inflammatory responses, such as BCR and TCR signaling pathway, cytokines, NK cytotoxicity, chemokines, IFN, IL, TGF-b and TNF family members, etc. The genes overlapping with aSAH_DEGs were defined as immunoinflammatory-related DEGs in aSAH. A total of 25 genes were obtained by overlapping the genes from the key WGCNA module, immune-related genes, and differentially expressed genes in aSAH, namely *IFI30*, *TYMP*, *VDR*, *LILRB3*, *IL10*, *OSM*, *C5AR1*, *CCR5*, *LYZ*, *FCER1G*, *CD1D*, *PLAUR*, *CXCR4*, *CRLF2*, *FPR2*, *CCRL2*, *SLC11A1*, *RNASE2*, *TNFSF8*, *FPR1*, *PIK3R5*, *AQP9*, *APOBEC3A*, *S100A8*, and *S100A9*. This gene set was defined as candidate immune-inflammatory genes associated with RNA methylation regulator-related transcriptional patterns in aSAH (Figure 3H).

### 3.5. Construction of RMRIGS Based on Integrated Machine Learning

Taken together, RNA methylation may function by influencing immune inflammation-related factors in aSAH, and inflammation-related genes are very important biomarkers for aSAH. Therefore, in order to explore the value of these genes, we accurately identified important RNA methylation-associated immunoinflammatory genes in aSAH by a machine learning approach. On this basis, we developed the RMRIGS for disease-status discrimination in aSAH, with the aim of improving clinical applicability. To further evaluate the diagnostic value of RNA methylation-related immune genes in aSAH, we applied an integrated machine learning framework to the 25 candidate genes. Feature selection results from the seven algorithms are summarized in Table 1.

**Figure 4:**
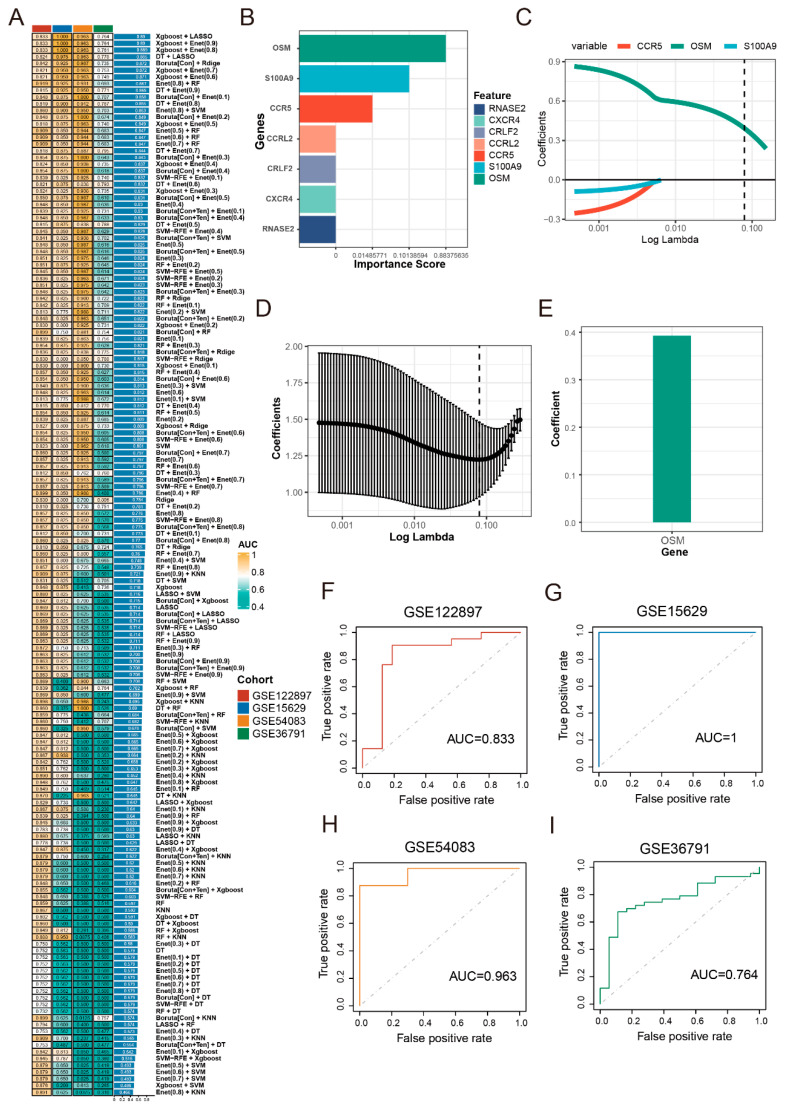
Identification of pivotal RM regulators in aSAH and construction of a diagnostic model. (**A**). Performance overview of 159 machine learning algorithm combinations based on AUC values across the training and validation datasets. (**B**). Importance ranking of Xgboost-based algorithms in GSE122897. (**C**). Parameter λ selection of the LASSO algorithms after cross-validation in GSE122897. (**D**). Different genes under various λ coefficient changes. (**E**). Coefficients of key variable *OSM* in LASSO. (**F**–**I**). ROC results of RMRIGS in predicting aSAH in training and validation datasets.

In the second step, 3-fold cross-validation was performed, and the model with the highest average AUC across the training cohort and external validation cohorts was selected (Figure 4A). The outcome variable can accurately discriminate aSAH from the control group, and the key variable of this model was extracted as RNA methylation-related immunoinflammatory genes (RMRIGS) in aSAH. Based on the combined XgBoost and LASSO algorithm, the model with the best average AUC value among the 159 combined algorithms was finally calculated. We found that the XgBoost algorithm identified three valuable genes, including OSM, CCR5, and S100A9, based on feature importance scores, when the significance score was taken to be >0 (Figure 4B). The XgBoost results were incorporated into the LASSO algorithm to obtain the final results (Figure 4C,D). After XGBoost-based feature prioritization, LASSO regression was applied to the selected candidate genes within the internal resampling framework. The final model retained oncostatin M (OSM) as the only variable, and the RMRIGS score was calculated as 0.0795 × *OSM* expression (Figure 4E). The above hyperparameter settings for machine learning are displayed in the Supplementary Tables S7–S11. The diagnostic performance of the OSM-based RMRIGS score was further evaluated using ROC analysis in the training and validation cohorts, showing good ability to discriminate aSAH samples from controls (Figure 4F–I). To further clarify model generalizability, the AUC, 95% confidence interval, sensitivity, and specificity for each dataset are summarized in Supplementary Table S12.

### 3.6. Mendelian Randomization Analysis Identifies OSM as a Causal Protective Factor for aSAH

**Figure 5:**
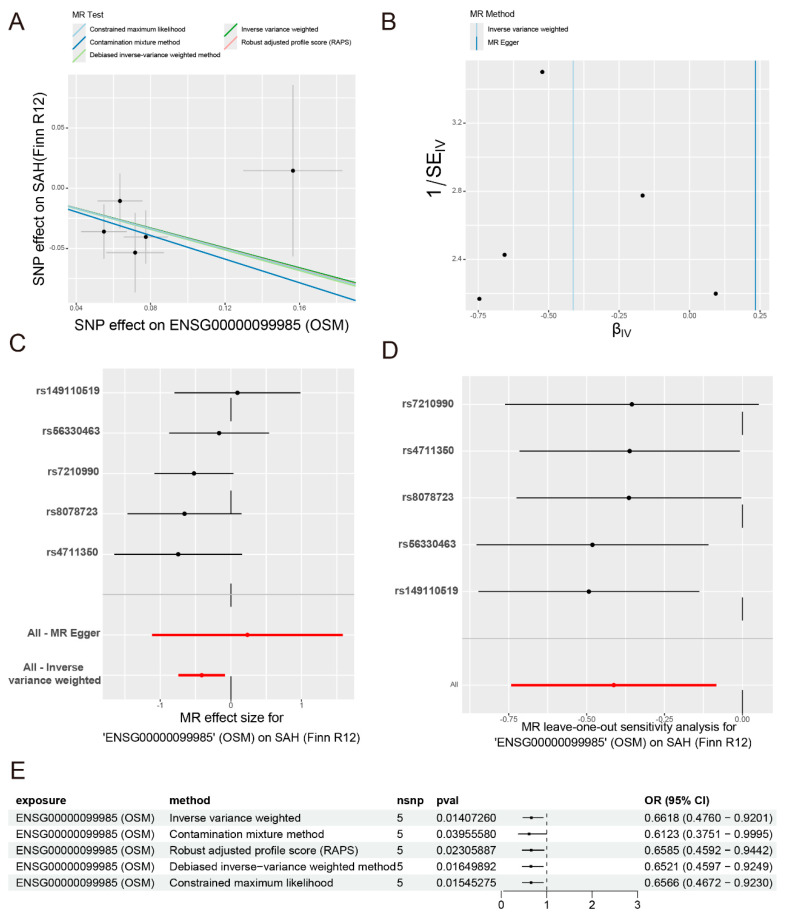
Mendelian randomization analysis of the causal association between OSM expression and SAH risk. (**A**). Scatter plot of SNP effects on OSM expression versus SAH risk, with fitted lines from different MR methods. (**B**). Funnel plot showing the distribution of SNP-specific causal estimates and the overall IVW and MR-Egger estimates. (**C**). Forest plot of SNP-specific and overall MR estimates for the effect of OSM on SAH risk. (**D**). Leave-one-out analysis assessing the influence of each individual SNP on the overall estimate. (**E**). Summary table of MR estimates obtained using IVW, ConMix, MR-RAPS, dIVW, and cML-MA. The results consistently supported a protective effect of genetically predicted OSM expression on SAH risk.

To further investigate the potential causal relationship between genetically predicted OSM expression and the risk of aneurysmal subarachnoid hemorrhage (aSAH), we performed a two-sample Mendelian Randomization (MR) analysis (Figure 5). Utilizing genetic variants as instrumental variables, the primary Inverse Variance Weighted (IVW) analysis revealed that higher expression of OSM was significantly associated with a decreased risk of aSAH (OR = 0.6618, 95% CI: 0.4760–0.9201, *p* = 0.014; Figure 5E). This protective association was also supported by alternative MR models, including the contamination mixture method (OR = 0.6123, *p* = 0.039), robust adjusted profile score (RAPS; OR = 0.6585, *p* = 0.023), and debiased inverse-variance weighted method (OR = 0.6521, *p* = 0.016).

The selected instrumental variables showed sufficient strength, with all F-statistics exceeding 10 (range: 20.08–42.39; mean: 29.23). No significant heterogeneity was detected by Cochran’s Q test using either IVW (Q = 2.721, *p* = 0.606) or MR-Egger regression (Q = 1.782, *p* = 0.619). In addition, no significant directional horizontal pleiotropy was detected by the MR-Egger intercept test (intercept = −0.0474, *p* = 0.404) or the MR-PRESSO global test (RSSobs = 3.961, *p* = 0.676). Detailed results of instrument strength, heterogeneity, and pleiotropy assessments are summarized in Supplementary Table S14.

Scatter plots and individual SNP effect estimates further demonstrated the robustness of this negative association (Figure 5A–C). Additionally, leave-one-out sensitivity analysis confirmed that the causal estimates were stable and not disproportionately influenced by any individual SNP (Figure 5D). Together, these results suggest a genetically supported protective association between OSM expression and SAH risk.

### 3.7. Biological Characteristics of High- and Low-RMRIGS Groups

**Figure 6:**
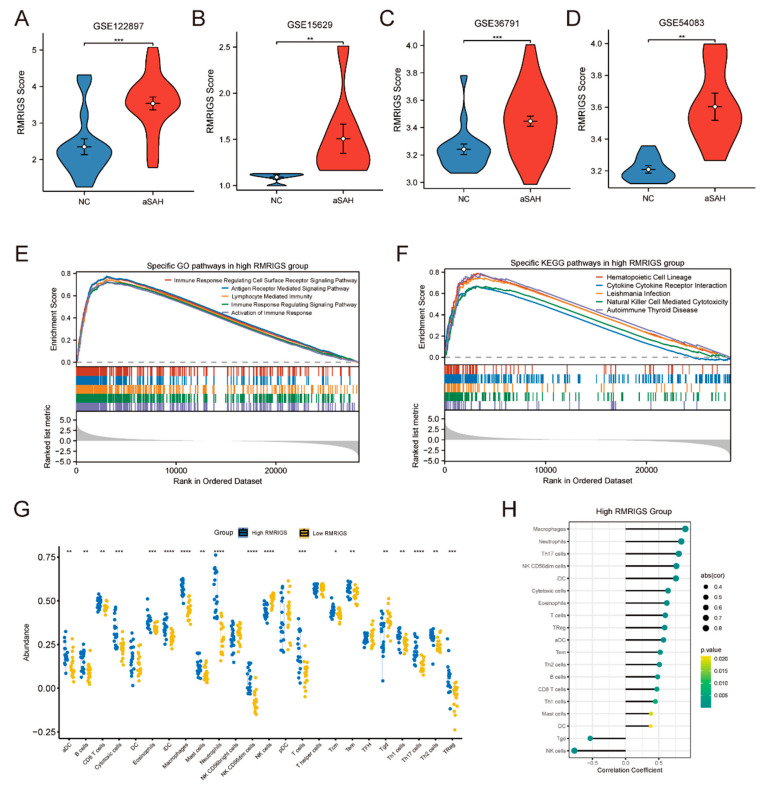
Evaluation of RMRIGS and biological functional enrichment and immune characterization in different scoring groups. (**A**–**D**). Comparison between high-RMRIGS and low-RMRIGS groups across different datasets. (**E**). GO enrichment results of GSEA in the high-RMRIGS group. (**F**). KEGG enrichment results of GSEA in the high-RMRIGS group. (**G**). Group comparison plot of the proportions of 24 immune cells using the ssGSEA algorithm. (**H**). Lollipop plot showing correlations between RMRIGS scores and estimated immune cell abundance. * *p*-value < 0.05, ** *p*-value < 0.01, *** *p*-value < 0.001, **** *p*-value < 0.0001.

To further investigate the potential biological mechanisms of RMRIGS, we divided all samples of GSE122897 into high-RMRIGS group and low-RMRIGS groups based on the median RMRIGS score and performed GSEA analysis. A concordance between RMRIGS and the occurrence of aSAH in different datasets could be clearly observed (Figure 6A–D). The high-RMRIGS group was enriched for many pathways associated with immune-inflammatory activation, such as those involved in immune response-regulating cell surface receptor signaling pathway, lymphocyte-mediated immunity, activation of an immune response, cytokine–cytokine receptor interaction, and so on (Figure 6E,F).

Using ssGSEA, we assessed the abundance of immune cells based on blood gene expression profiles and identified 24 immune cells with differential infiltration in the high-RMRIGS group (Figure 6G). Remarkably, the high-RMRIGS group exhibited a higher abundance of myeloid cells (e.g., macrophages and neutrophils) but fewer lymphocytes (e.g., NK cells, natural immune T cells). The high-RMRIGS group showed a strong positive correlation with macrophages and neutrophilic clusters and a negative correlation with NK cells (Figure 6H), suggesting that the RMRIGS can capture the peripheral blood immune cell change in aSAH patients. In summary, patients with high RMRIGS scores exhibited more pronounced immune-inflammatory alterations, suggesting that a high RMRIGS score may reflect stronger disease-associated immune-inflammatory activity in established aSAH samples.

### 3.8. Single-Cell Profiling Reveals Cell Type Distribution and Osm Enrichment After SAH

**Figure 7:**
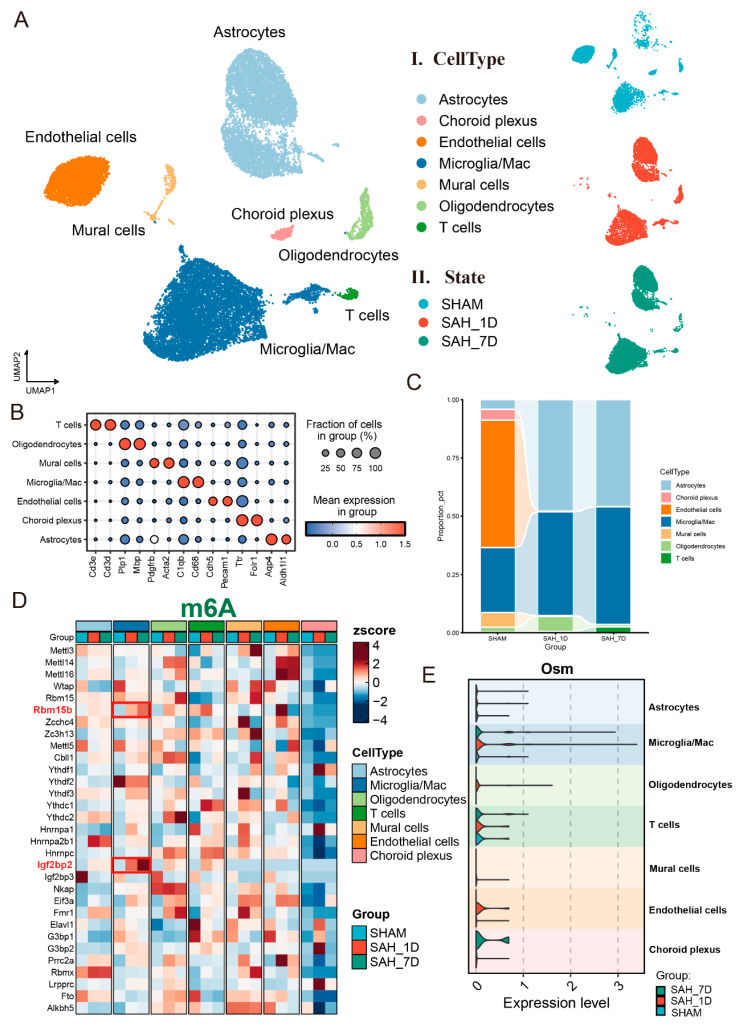
Single-cell RNA-seq analysis showing the cellular landscape, m6A regulator expression, and *Osm* distribution in sham and SAH mouse brains. (**A**). UMAP plot showing the major cell types in the integrated single-cell dataset, colored by cell type and group. (**B**). Dot plot of representative marker genes used for cell type annotation. Dot size indicates the fraction of expressing cells, and color indicates the average expression level. (**C**). Proportions of major cell types across the SHAM, SAH_1D, and SAH_7D groups. (**D**). Heatmap showing the expression patterns of m6A regulators across major cell types and groups. (**E**). Violin plot showing *Osm* expression across major cell populations.

To characterize the cellular landscape after SAH and identify the potential source of *Osm*, we analyzed scRNA-seq data from sham and SAH mouse brains (Figure 7). UMAP clustering resolved seven major cell populations, including astrocytes, choroid plexus cells, endothelial cells, microglia/macrophages, mural cells, oligodendrocytes, and T cells (Figure 7A). The identities of these populations were confirmed by the expression of canonical marker genes, as shown in the dot plot (Figure 7B). Comparison of cell composition across groups further revealed marked shifts in the post-SAH microenvironment, with a clear increase in the proportion of microglia/macrophages after SAH, accompanied by relative changes in several non-myeloid cell populations (Figure 7C). We next examined the distribution of m6A regulators across the major cell types and found that these regulators displayed distinct cell type- and state-specific expression patterns, with several molecules showing relatively prominent changes in the microglia/macrophage compartment (Figure 7D). Importantly, *Osm* expression was not broadly distributed across all cell types but was mainly enriched in microglia/macrophages and was elevated after SAH (Figure 7E). These findings suggested that myeloid cells, particularly the microglia/macrophage lineage, may represent the principal cellular source of *Osm* in the SAH microenvironment and further supported our bulk transcriptomic observation that myeloid-related immune infiltration is a major feature of SAH. This analysis was designed to complement the human bulk transcriptomic findings by resolving the potential cellular source and recipient populations of OSM-related signaling after SAH, rather than to directly validate the human diagnostic model.

### 3.9. Single-Cell Analysis Suggests a Macrophage-Derived Osm-Associated Communication Axis Targeting Microglia

**Figure 8:**
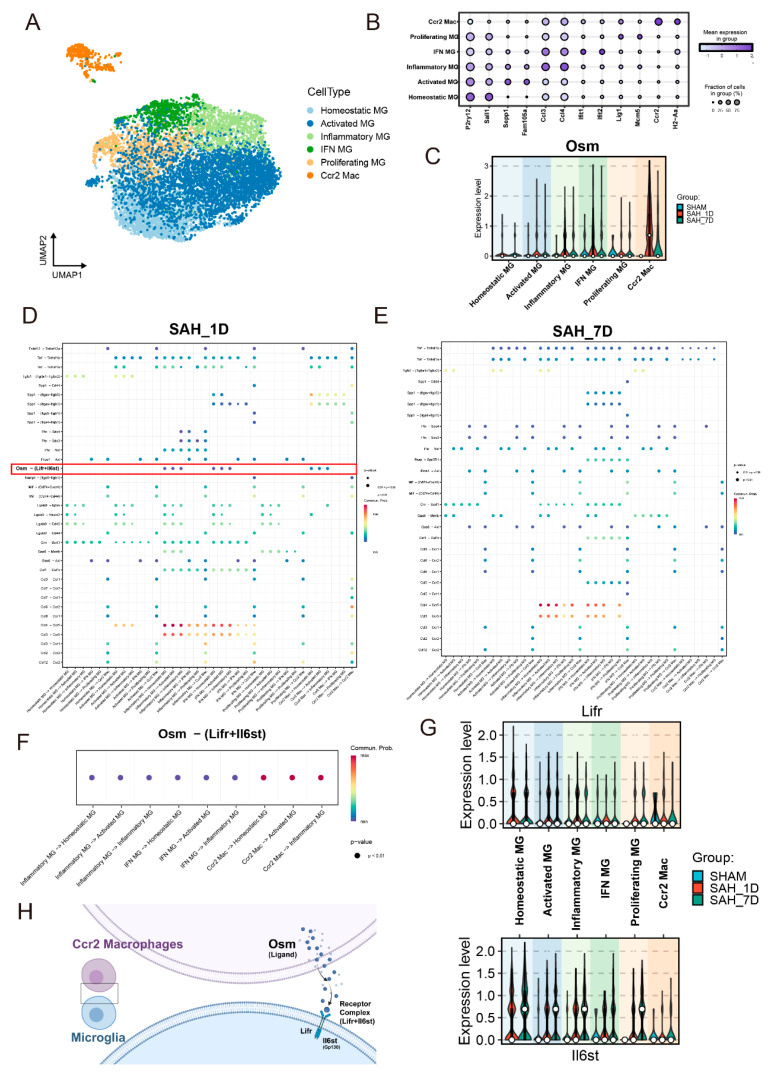
Single-cell analysis of the myeloid compartment suggests a potential Osm-associated intercellular communication pattern in SAH. (**A**). UMAP plot showing the major myeloid subclusters, including homeostatic microglia, activated microglia, inflammatory microglia, IFN microglia, proliferating microglia, and *Ccr2* macrophages. (**B**). Dot plot of representative marker genes used for myeloid subcluster annotation. Dot size indicates the fraction of expressing cells, and color indicates the average expression level. (**C**). Violin plot showing *Osm* expression across myeloid subclusters and groups. (**D**). CellChat analysis of ligand–receptor interactions among myeloid subclusters in the SAH_1D group. (**E**). CellChat analysis of ligand–receptor interactions among myeloid subclusters in the SAH_7D group. (**F**). Bubble plot showing *Osm* − (*Lifr* + *Il6st*) signaling interactions among myeloid subclusters. (**G**). Violin plots showing *Lifr* and *Il6st* expression across myeloid subclusters and groups. (**H**). Schematic diagram illustrating the proposed Osm-mediated communication axis between *Ccr2* macrophages and microglia.

Reclustering of the microglia/macrophage population identified six transcriptionally distinct subclusters, including homeostatic microglia, activated microglia, inflammatory microglia, IFN microglia, proliferating microglia, and infiltrating *Ccr2* macrophages (Figure 8A). The identities of these subclusters were determined according to established marker genes and further supported by their corresponding expression patterns (Figure 8B). Among these subpopulations, *Osm* expression was mainly enriched in the *Ccr2* macrophage cluster and was most prominent at the early stage after SAH, especially in the SAH_1D group (Figure 8C), suggesting that infiltrating macrophages may represent the major source of Osm after hemorrhagic injury. In contrast, the receptor component genes *Lifr* and *Il6st* were broadly expressed across several microglial subclusters rather than being confined to a single population (Figure 8G), indicating that resident microglia may serve as the main recipient cells of OSM signaling. In line with this, CellChat analysis detected the *Osm* − (*Lifr* + *Il6st*) signaling axis in both the SAH_1D and SAH_7D groups (Figure 8D,E). Communication probability analysis further showed that *Ccr2* macrophages were the principal OSM-sending cells, whereas multiple microglial subclusters, particularly activated, inflammatory, and IFN microglia, acted as potential receivers of this signal (Figure 8F). Collectively, these findings suggest a potential macrophage-derived Osm-associated communication axis targeting microglia after SAH and provide a cellular basis for the inflammatory signaling pattern identified in the human bulk transcriptomic analysis.

### 3.10. Validation of the OSM-LIFR/GP130 Signaling Axis and Its Association with RNA Methylation in aSAH

**Figure 9:**
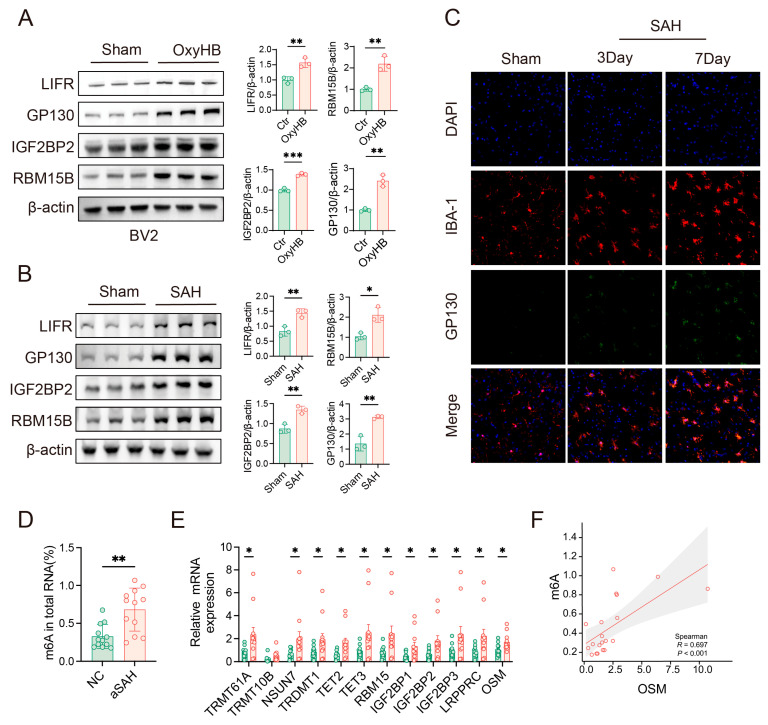
Experimental and clinical validation of the OSM-related receptor axis and m6A-associated alterations in SAH. (**A**). Western blot analysis and quantification of LIFR protein, GP130 protein encoded by IL6ST, IGF2BP2, and RBM15B expression in BV2 cells after OxyHB treatment. β-actin was used as the loading control. (**B**). Western blot analysis and quantification of LIFR protein, GP130 protein encoded by *IL6ST*, IGF2BP2, and RBM15B expression in brain tissues from sham and SAH mice. β-actin was used as the loading control. (**C**). Immunofluorescence staining of IBA1 and GP130 in sham and SAH brain tissues at different time points, showing increased GP130 expression and colocalization with IBA1-positive cells after SAH. (**D**). Comparison of total m6A RNA levels in peripheral blood samples between normal controls and patients with aSAH. (**E**). Relative mRNA expression of selected RNA methylation-related genes and *OSM* in peripheral blood samples from normal controls and patients with aSAH. (**F**). Correlation analysis showing a positive association between *OSM* expression and total m6A RNA levels in peripheral blood samples from patients with aSAH. * *p*-value < 0.05, ** *p*-value < 0.01, *** *p*-value < 0.001.

To further assess the expression status of the OSM receptor axis, we performed in vitro and in vivo experiments focusing on LIFR protein encoded by *LIFR* and GP130 protein encoded by *IL6ST*, as well as their association with RNA methylation-related regulators (Figure 9). In BV2 microglial cells treated with OxyHB to simulate the aSAH environment, Western blot analysis revealed that the protein expression levels of LIFR and GP130 were significantly increased compared with those in the control group. Notably, the RNA methylation-related regulators IGF2BP2 and RBM15B were also markedly upregulated under the same conditions (Figure 9A). These findings were consistently reproduced in brain tissues from the SAH mouse model, in which LIFR, GP130, IGF2BP2, and RBM15B all showed significantly increased expression relative to the sham group (Figure 9B). Immunofluorescence staining further demonstrated that GP130 expression was elevated in IBA1-positive microglia, with a notable increase at three and seven days after SAH induction, suggesting activation of the OSM receptor complex in resident immune cells (Figure 9C). Furthermore, we extended the validation to clinical samples to confirm the relevance of these findings in human patients. Consistent with our bioinformatic predictions, total m6A methylation levels were significantly elevated in the peripheral blood of patients with aSAH compared with healthy controls (Figure 9D). Quantitative PCR analysis showed that *OSM* and selected RNA methylation regulators were upregulated in the aSAH group (Figure 9E). Finally, Spearman correlation analysis showed a strong positive association between *OSM* expression and the global level of m6A methylation (R = 0.697, *p* < 0.001) (Figure 9F). This correlation was interpreted as an association only and did not indicate that OSM expression was directly regulated by m6A modification or that OSM signaling causally regulated RNA methylation. To further examine whether m6A regulator-expression patterns were related to directly measured m6A levels, we calculated an m6A-regulator expression score using the available qRT-PCR-measured m6A regulators, including RBM15, IGF2BP1, IGF2BP2, IGF2BP3, and LRPPRC. This score was positively correlated with total m6A levels in the same peripheral blood samples (Spearman R = 0.515, *p* = 0.0099; Supplementary Table S16). Effect-size estimation and post hoc power analysis were also performed for total m6A levels and qRT-PCR validation markers, with the results summarized in Supplementary Table S17.

## 4. Discussion

The immune-inflammatory response is a cornerstone of the pathological progression of aSAH, influencing every stage from initial aneurysm rupture to the development of EBI [27], cerebral vasospasm (CVS) [28], and DCI [29]. In this study, we explored RNA methylation regulator-related transcriptional patterns associated with immune-inflammatory alterations in aSAH. By integrating machine learning, Mendelian randomization, single-cell analysis, and experimental validation, we identified OSM as an immune-inflammatory feature within this analytical framework and inferred a potential Osm-associated communication pattern between infiltrating macrophages and resident microglia.

Therefore, probing the molecular mechanisms involved in the immune response and other processes mediated by it will help us to explore new ideas for the prevention and diagnosis of aSAH. RNA methylation, a modification that regulates genes at the post-transcriptional and translational levels, mediates the biological processes of a wide range of diseases. The most common of these, m6A modification, has been reported by several studies to be strongly associated with inflammation. m6A-associated proteins, such as METTL3, METTL14, and IGF2BP2, can regulate or recognize changes in m6A levels to mediate inflammatory responses [30,31,32], or they can directly regulate the level of m6A modifications in inflammatory factors or immune-related proteins [33]. Inflammatory responses can also modulate the expression of m6A-related proteins as well as influence m6A levels [34,35]. In addition to m6A, there are new types of RNA methylation such as m5C, m1A, and m7G. RNA methylation is dynamically regulated by specific methyltransferases (Writer) and demethylases (Eraser), which cooperate to maintain the proper mRNA methylation state, and methylation recognition proteins (Reader), which recognize and bind to methylated mRNAs, thereby regulating mRNA transcription and translation [12].

To identify immune-inflammatory genes associated with RNA methylation regulator-related transcriptional patterns in aSAH, we first analyzed the expression of “Writer”, “Reader” and “Eraser” genes regulating RNA methylation in the aSAH dataset and found that the expression levels of various regulatory proteins of m6A, m5C, and m1A were significantly altered in aSAH. Subsequently, we used ssGSEA to calculate enrichment scores for m6A, m5C, and m1A for each sample and screened the set of modular genes associated with the three RNA methylations by WGCNA. We found that modular genes were mostly enriched in immune-inflammation-related pathways by GO and KEGG enrichment analysis of the modular gene sets. Therefore, we suggested that RNA methylation was closely associated with immune inflammation in aSAH and that RNA methylation-associated immune-associated genes, RMRIGs, may be new biomarkers for aSAH. Subsequently, we obtained 25 RNA methylation-associated immunoinflammation-associated DEGs by differential analysis of differentially expressed genes (DEGs) in the NC and aSAH groups, and then took the intersection with RNA methylation-associated modular genes. Based on 159 combinations of nine machine learning algorithms, the combination of XgBoost and Lasso was determined to be the optimal algorithm to most effectively discriminate between the NC group and the aSAH group, suggesting that *OSM* was the key feature retained in the final RMRIGS model and may reflect disease-associated immune-inflammatory activity in aSAH.

This interpretation should also consider the practical limitations of human aSAH sampling. Given the difficulty of obtaining ruptured human aneurysm wall tissue and appropriate normal intracranial arterial controls, the public bulk transcriptomic datasets, mouse single-cell data, and peripheral blood samples used here were interpreted as complementary evidence rather than direct substitutes for aneurysm wall pathology. At present, considerable effort has been devoted to identifying biomarkers for aSAH, not only to improve the prediction of disease occurrence, complications, and poor outcomes, but also to provide a reference for treatment timing through dynamic changes in key molecules [36]. Previous studies have reported a variety of potential biomarkers, including blood components, cytokines, microRNAs, and electrolytes [37]. Recent reviews have further summarized molecular targets involved in intracranial aneurysm treatment, including inflammatory mediators, extracellular matrix remodeling, oxidative stress, endothelial dysfunction, and vascular smooth muscle cell phenotypic modulation [38]. In addition, microRNAs have been proposed as potential biomarkers and regulatory molecules for SAH diagnosis, prognosis, and therapeutic development [39]. These studies highlight the increasing importance of molecularly defined signatures in intracranial aneurysm and SAH research, while also emphasizing the need for further validation before clinical translation. In the field of RNA methylation, Yuan et al. reported that the m6A regulator WTAP may participate in intracranial aneurysm-related processes by influencing the expression status and m6A modification level of brain microvascular endothelial cells [40], highlighting the potential importance of RNA methylation in cerebrovascular diseases. In contrast, the samples included in our study were more focused on the clinical condition of aneurysm rupture with hemorrhage. Therefore, compared with previous studies centered on unruptured aneurysms or other disease stages, some RNA molecules did not show fully consistent differential patterns in our datasets. Notably, in our training cohort, OSM exhibited a more prominent expression change than several molecules reported in previous studies (Supplementary Table S13) [41,42]. In addition, the signature developed in this study maintained good discriminatory performance in external datasets, suggesting a certain degree of cross-cohort stability. Compared with previously reported RNA methylation regulator-related signatures, although some models performed better in their own training cohorts, our model showed more stable performance in independent validation datasets, which may indicate better generalizability [17].

Inflammation is a key issue in aSAH, but the inflammatory response itself is highly stage-dependent and bidirectional, potentially contributing both to tissue injury and to subsequent repair [43]. Accordingly, understanding its temporal dynamics and identifying appropriate therapeutic windows remain major challenges. As a member of the IL-6 cytokine family, OSM has been shown to exert pro-inflammatory or immunomodulatory effects under different microenvironmental conditions [44]. Previous studies have demonstrated that OSM can mediate inflammatory responses through activation of the JAK/STAT pathway [33]. In aSAH-related research, OSM has also been reported to participate in ferroptosis and early brain injury by regulating the expression of Hepc, DMT1, and FPN1[45]. On the other hand, some studies have suggested a potential neuroprotective role of OSM. For example, Weiss et al. found that OSM attenuated excitotoxic cell death both in vitro and in vivo [46]. These apparently divergent observations suggest that the biological effects of OSM may be highly dependent on disease stage and the surrounding microenvironment. In the context of our findings, OSM may be better understood as a key immune-inflammatory node associated with RNA methylation regulator-related transcriptional patterns in aSAH. Although the current evidence is insufficient to demonstrate that OSM is directly regulated by RNA methylation, its association with global m6A levels and multiple methylation-related molecules suggests a link that warrants further investigation.

At present, increasing efforts have been devoted to identifying biomarkers for aSAH risk stratification and prognosis, but most candidate markers remain limited to associative evidence rather than causal inference. In the present study, we further incorporated two-sample Mendelian randomization analysis to evaluate the relationship between OSM and SAH risk at the genetic level. The results showed that genetically predicted higher *OSM* expression was associated with a lower risk of SAH (OR = 0.6618, *p* = 0.014), supporting a potential protective association of OSM in disease occurrence. At the same time, the RMRIGS developed in this study showed good discriminatory performance in both the training cohort and external validation cohorts, suggesting that this OSM-centered RMRIGS may have potential value for disease-status assessment in aSAH. However, these findings still require further validation in larger samples and prospective cohorts. It should be noted that the RMRIGS score was derived from case–control transcriptomic datasets and should be interpreted as a disease-associated diagnostic expression score rather than a prospective risk score. In established aSAH samples, increased *OSM* expression may reflect acute post-rupture immune-inflammatory activation, myeloid cell infiltration, or compensatory signaling. By contrast, MR evaluates the association between genetically predicted OSM expression and SAH risk over a longer biological time scale. Therefore, the high-RMRIGS group showing stronger inflammatory enrichment does not necessarily contradict the protective association observed in MR analysis.

The neuroinflammatory response in aSAH is characterized by substantial cellular heterogeneity, which represents a major challenge in understanding its immune-pathological mechanisms. Through single-cell transcriptomic analysis, we further resolved the cellular composition and signaling context of the post-SAH brain and found that Osm was mainly enriched in infiltrating *Ccr2*+ macrophages, whereas its receptor component genes *Lifr* and *Il6st* were more broadly distributed across multiple microglial subclusters, particularly activated and inflammatory microglia. Further cell–cell communication analysis showed that the *Osm* − (*Lifr* + *Il6st*) signaling axis was markedly enhanced after SAH, suggesting that infiltrating peripheral macrophages may influence the inflammatory state of resident microglia through paracrine signaling. To some extent, these findings provide a cellular explanation for our bulk transcriptomic observation that high RMRIGS scores were closely associated with myeloid immune cell infiltration, and they also suggest a potential link between peripheral immune activation and local neuroinflammation after SAH.

From a translational perspective, blood-based biomarkers are more feasible for clinical application than tissue-derived indicators. In the present study, validation using peripheral blood samples showed that total m6A levels were significantly elevated in patients with aSAH compared with healthy controls. In addition, *OSM* and multiple RNA methylation-related molecules were significantly upregulated in the aSAH group, and *OSM* expression was positively correlated with total m6A levels. These findings were broadly consistent with our bioinformatic analyses and further supported an association between m6A-related changes, RNA methylation regulator expression, and immune-inflammatory activation in aSAH. Moreover, the observed upregulation of LIFR, GP130, IGF2BP2, and RBM15B in the SAH models suggested that activation of OSM receptor axis-related proteins occurred together with changes in m6A-regulator expression under SAH-related conditions. However, these data should not be interpreted as evidence that OSM is directly regulated by RNA methylation or that OSM signaling causally controls RNA methylation. Rather, *OSM* was identified within an immune-inflammatory gene set associated with RNA methylation regulator-related transcriptional patterns, and its correlation with total m6A levels indicates an association only. Direct mechanistic studies, including transcript-specific m6A profiling of *OSM* transcripts, perturbation of m6A writers/readers, and OSM-pathway intervention experiments, are required to determine whether a causal regulatory relationship exists.

Several limitations should be considered when interpreting the present findings. Although this study integrated public transcriptomic datasets, single-cell data, experimental models, and clinical validation samples, these data sources represent different biological contexts rather than directly matched pathological materials. The public bulk transcriptomic datasets provided useful disease-associated expression patterns, but they could not fully resolve cell type heterogeneity or control for clinical factors such as disease phase, hypertension status, medication, emergency treatment, and longitudinal outcome. Moreover, direct analysis of ruptured human aneurysm wall tissue or matched post-rupture brain tissue remains difficult in clinical practice; therefore, the present findings should be viewed as exploratory molecular clues rather than definitive evidence of aneurysm wall pathology. Similarly, the mouse single-cell data, BV2 cells, and mouse SAH models helped characterize potential cellular sources and receptor axis changes, but they cannot fully recapitulate the complex process of human saccular aneurysm formation, rupture, and post-aSAH injury.

Another important consideration is that the molecular scores and associations reported here should not be overinterpreted as direct causal mechanisms. The RMRIGS model was derived from case–control transcriptomic datasets and therefore reflects a disease-associated diagnostic expression pattern rather than a prospective risk-prediction model for future aneurysm rupture or SAH onset. In addition, the ssGSEA-derived RNA methylation regulator-related scores were based on regulator mRNA expression and cannot directly measure RNA methylation abundance or enzymatic activity. Although the clinical m6A-regulator expression score was correlated with directly measured total m6A levels, this expression-based index cannot substitute for direct RNA methylation profiling. Finally, the present study did not functionally verify whether RNA methylation directly regulates *OSM* expression or whether the inferred OSM–LIFR/GP130 communication axis causally drives microglial activation. Further studies using larger prospective cohorts, human disease-relevant samples, transcript-specific m6A profiling, OSM pathway blockade, and *LIFR* or *IL6ST*/GP130 perturbation are needed to clarify these mechanisms.

## 5. Conclusions

In summary, this study characterized RNA methylation regulator-related immune dysregulation in aSAH through integrated transcriptomic, Mendelian randomization, single-cell, experimental, and clinical validation analyses. We identified an OSM-centered RMRIGS with consistent discriminatory performance across multiple cohorts. Single-cell analysis suggested that *Osm* was mainly enriched in infiltrating *Ccr2*+ macrophages, whereas *Lifr* and *Il6st* were broadly expressed in microglial subpopulations, indicating a potential OSM-related macrophage-to-microglia communication pattern after SAH. In parallel, increased total m6A levels and upregulation of OSM-associated receptor axis proteins and RNA methylation regulators in experimental and clinical samples supported an association between immune-inflammatory activation and m6A-related alterations in aSAH. These findings provide candidate molecular clues for understanding post-SAH neuroinflammation and suggest that the OSM–LIFR/GP130 axis may represent a potential direction for future mechanistic and translational studies. However, the present study does not establish a direct causal relationship between OSM signaling and RNA methylation, which requires further experimental validation.

## Figures and Tables

**Table 1:** Machine learning feature selection results in aSAH.

Algorithms	Selected Genes	Number
LASSO	*IFI30*	1
Ridge	*IFI30*, *TYMP*, *VDR*, *LILRB3*, *IL10*, *OSM*, *C5AR1*, *CCR5*, *LYZ*, *FCER1G*, *CD1D*, *PLAUR*, *CXCR4*, *CRLF2*, *FPR2*, *CCRL2*, *SLC11A1*, *RNASE2*, *TNFSF8*, *FPR1*, *PIK3R5*, *AQP9*, *APOBEC3A*, *S100A8*, *S100A9*	25
Enet[alpha = 0.1]	*IFI30*, *TYMP*, *VDR*, *LILRB3*, *IL10*, *OSM*, *C5AR1*, *CCR5*, *LYZ*, *FCER1G*, *CD1D*, *PLAUR*, *CXCR4*, *CRLF2*, *FPR2*, *CCRL2*, *SLC11A1*, *RNASE2*, *TNFSF8*, *AQP9*, *APOBEC3A*	21
Enet[alpha = 0.2]	*IFI30*, *TYMP*, *VDR*, *LILRB3*, *IL10*, *OSM*, *C5AR1*, *CCR5*, *LYZ*, *CD1D*, *PLAUR*, *CXCR4*, *CRLF2*, *FPR2*, *CCRL2*, *SLC11A1*, *RNASE2*, *TNFSF8*, *APOBEC3A*	19
Enet[alpha = 0.3]	*IFI30*, *TYMP*, *VDR*, *LILRB3*, *IL10*, *OSM*, *C5AR1*, *CCR5*, *LYZ*, *CRLF2*, *FPR2*	10
Enet[alpha = 0.4]	*IFI30*, *TYMP*, *VDR*, *LILRB3*, *IL10*, *OSM*, *C5AR1*	7
Enet[alpha = 0.5]	*IFI30*, *VDR*, *LILRB3*, *IL10*, *OSM*	5
Enet[alpha = 0.6]	*IFI30*, *VDR*, *LILRB3*, *IL10*, *OSM*	5
Enet[alpha = 0.7]	*IFI30*, *VDR*, *LILRB3*, *IL10*, *OSM*	5
Enet[alpha = 0.8]	*IFI30*, *VDR*, *IL10*, *OSM*	4
Enet[alpha = 0.9]	*IFI30*, *IL10*	2
Boruta[Con]	*LILRB3*, *IL10*, *OSM*, *IFI30*, *SLC11A1*	5
Boruta[Con + Ten]	*IFI30*, *TYMP*, *VDR*, *LILRB3*, *IL10*, *OSM*, *CCR5*, *FCER1G*, *CXCR4*, *SLC11A1*, *AQP9*	11
SVM-RFE	*OSM*, *LILRB3*, *IL10*, *FCER1G*, *AQP9*, *IFI30*, *CXCR4*, *VDR*, *C5AR1*, *PIK3R5*, *SLC11A1*, *CCR5*, *TYMP*, *CD1D*, *RNASE2*, *S100A8*, *FPR2*, *FPR1*, *APOBEC3A*, *LYZ*, *S100A9*, *CCRL2*, *PLAUR*	23
RF	*IL10*, *OSM*, *IFI30*, *FCER1G*	4
Xgboost	*OSM*, *S100A9*, *CCR5*	3
Decision Tree	*OSM*, *CCR5*, *TYMP*, *CXCR4*, *CD1D*, *C5AR1*	6

## Data Availability

The datasets used in this study were obtained from the Gene Expression Omnibus (GEO) database (http://www.ncbi.nlm.nih.gov/geo, 20 November 2025) and the OMIX database (https://ngdc.cncb.ac.cn/, 25 November 2025). Summary-level data for Mendelian randomization analysis were derived from publicly available genome-wide association study datasets.

## Supplementary Materials

The following supporting information can be downloaded at: https://www.mdpi.com/article/10.3390/biomedicines14061254/s1.
